# Implementing multifactorial psychotherapy research in online virtual environments (IMPROVE-2): study protocol for a phase III trial of the MOST randomized component selection method for internet cognitive-behavioural therapy for depression

**DOI:** 10.1186/s12888-016-1054-8

**Published:** 2016-10-06

**Authors:** Edward Watkins, Alexandra Newbold, Michelle Tester-Jones, Mahmood Javaid, Jennifer Cadman, Linda M. Collins, John Graham, Mohammod Mostazir

**Affiliations:** 1 Sir Henry Wellcome Building for Mood Disorders Research, College of Life and Environmental Sciences, University of Exeter, Exeter, EX4 4QG UK; 2 The Methodology Center and Department of Human Development and Family Studies, The Pennsylvania State University, Health and Human Development Building, University Park, PA 16802 USA

**Keywords:** Online, Cognitive behavioural therapy, Depression, MOST, Factorial

## Abstract

**Background:**

Depression is a global health challenge. Although there are effective psychological and pharmaceutical interventions, our best treatments achieve remission rates less than 1/3 and limited sustained recovery. Underpinning this efficacy gap is limited understanding of how complex psychological interventions for depression work. Recent reviews have argued that the active ingredients of therapy need to be identified so that therapy can be made briefer, more potent, and to improve scalability. This in turn requires the use of rigorous study designs that test the presence or absence of individual therapeutic elements, rather than standard comparative randomised controlled trials. One such approach is the Multiphase Optimization Strategy, which uses efficient experimentation such as factorial designs to identify active factors in complex interventions. This approach has been successfully applied to behavioural health but not yet to mental health interventions.

**Methods/Design:**

A Phase III randomised, single-blind balanced fractional factorial trial, based in England and conducted on the internet, randomized at the level of the patient, will investigate the active ingredients of internet cognitive-behavioural therapy (CBT) for depression. Adults with depression (operationalized as PHQ-9 score ≥ 10), recruited directly from the internet and from an UK National Health Service Improving Access to Psychological Therapies service, will be randomized across seven experimental factors, each reflecting the presence versus absence of specific treatment components (activity scheduling, functional analysis, thought challenging, relaxation, concreteness training, absorption, self-compassion training) using a 32-condition balanced fractional factorial design (2_IV_^7-2^). The primary outcome is symptoms of depression (PHQ-9) at 12 weeks. Secondary outcomes include symptoms of anxiety and process measures related to hypothesized mechanisms.

**Discussion:**

Better understanding of the active ingredients of efficacious therapies, such as CBT, is necessary in order to improve and further disseminate these interventions. This study is the first application of a component selection experiment to psychological interventions in depression and will enable us to determine the main effect of each treatment component and its relative efficacy, and cast light on underlying mechanisms, so that we can systematically enhance internet CBT.

**Trial registration:**

Current Controlled Trials ISRCTN24117387. Registered 26 August 2014

**Electronic supplementary material:**

The online version of this article (doi:10.1186/s12888-016-1054-8) contains supplementary material, which is available to authorized users.

## Background

Depression is a major global health challenge [1, 2]: it is highly prevalent, recurrent [3] and a leading cause of disability worldwide, with enormous individual, societal, and economic burden [4]. Although both antidepressant medication and cognitive-behavioural therapy (CBT) are evidence-based and recommended treatments for depression, there is still a major treatment gap. First, even these, our best treatments, only achieve remission rates of less than 1/3 and limited sustained recovery (50–80 % relapse/recurrence) [5] (an *efficacy gap*). Second, there is sub-optimal adherence to antidepressants and patients express a preference for psychological interventions (a *preference gap*). Third, traditional face-to-face psychotherapy can never be sufficiently widely available to reduce the global burden of depression, indicating the need for alternative delivery methods [5, 6] (a *scalability gap*).

One solution to address the scalability gap is via internet-based or e-therapy. Internet-CBT for depression has comparable efficacy to face-to-face treatment when supported by a therapist [7], can increase access by removing time constraints and geographical restrictions to attending therapy [7], and can increase cost-effectiveness and treatment coverage [8] by reducing therapist treatment time. Effective unguided e-therapy would have almost no constraints on volume of use and would be globally scalable and accessible.

Underpinning the efficacy gap is limited understanding of how complex psychological interventions for depression work. Resolving the active mechanisms of psychological interventions has been identified as a major priority [5, 9–11]. Uncovering the mechanisms of psychological treatments and optimising the content and delivery of treatments is necessary to build more potent, scalable, and efficient treatments. Because little progress has been made in specifying the active ingredients of CBT for depression [5, 9, 10], there have been no significant gains in the effectiveness of CBT for depression for over 30 years. Recent reviews have argued that we need to identify active elements so that therapy can be parsed and distilled to focus on what is essential and most engaging to patients, making it briefer, more potent, and facilitating wider dissemination and coverage [11, 12]. The Institute of Medicine recently proposed that determination of which elements are critical depends on testing the presence or absence of individual therapeutic elements in rigorous study designs [12].

One reason for limited progress in understanding mechanisms is the overreliance on parallel group comparative randomised controlled trials (RCTs). Such RCTs are effective for establishing the relative efficacy of one treatment intervention versus another, where they provide the gold standard for determining if an intervention works. However, they are limited at investigating the specific mechanisms of how interventions work because they can only compare the overall effects of each intervention package. Psychological treatments such as CBT are complex interventions, typically made up of multiple elements and components, each of which potentially acts via distinct mechanisms.

In standard comparative RCTs, all treatment components and their closely related hypothetical mechanisms are aggregated and confounded together in the comparison of treatment conditions. As a consequence, this design is unable to test specific main effects of treatment components and any possible synergistic or antagonistic interactions between individual treatment components, limiting advances in mechanistic understanding. If an RCT finds one treatment better than another, we don’t know which components made a difference; if there is no difference, we don’t know whether there are any components that effected an improvement.

Instead, we propose an innovative approach to begin to address both the scalability and efficacy gaps by using a fractional factorial design for the first time to identify the active ingredients within internet-delivered CBT for depression (the IMPROVE-2 study; Implementing Multifactorial Psychotherapy Research in Online Virtual Environments). This approach directly responds to the Institute of Medicine’s (2015) call for rigorous trial design to disentangle specific therapy elements.

Our approach is a theory-driven component selection experiment framed within the Multiphase Optimization Strategy (MOST) [13–19] approach. MOST, rooted in engineering, agriculture, and behavioural science, is a principled and comprehensive framework for optimizing and evaluating behavioural interventions [13–15].

The optimization phase of MOST uses efficient experimentation to identify which of a set of candidate components is effective and should be included in the optimized intervention. Although a wide variety of experimental designs are possible candidates for use in the optimization phase, frequently factorial or fractional factorial designs emerge as most efficient [14, 15, 20]. Factorial and fractional factorial experiments allow one to explore main effects of factors and interactions among factors [20]. A fractional factorial design is a variation on the factorial design that employs a systematic approach to reduce the number of experimental conditions to allow a more manageable study, at the cost of allowing only main effects and a pre-specified set of interactions to be tested. Fractional factorial designs require the assumption that higher-order interactions are negligible in size, because they are combined, or aliased, with lower-order effects. In the evaluation phase of MOST, the optimized intervention, constructed based on the findings from the optimization phase and assembled to meet a specific optimization criterion, is compared against a control intervention in a standard RCT.

Critically, the optimization phase proposed by MOST [13] provides direct evidence about the effects and interactions of individual components within a treatment package, which is necessary for methodically enhancing and simplifying complex interventions [21]. This approach enables us to develop a mechanistic understanding of therapy and to select active and reject inactive/counter-productive components or elements.

MOST is well-validated [13, 22–25] and recommended within the Medical Research Council Complex Intervention guidelines [26]. A key advantage is greater experimental efficiency, with a focus on identifying “active ingredients” versus “inactive” or extraneous components before moving onto large-scale comparative trials, resulting in fewer overall resources required to answer the research questions in the long run than with the traditional approach [27]. However, to date, MOST has not been applied to psychological interventions for mental health.

In the current study, we therefore will combine the MOST approach with an e-health delivery format for CBT to build in treatment reach, scalability, and increased treatment coverage for the optimised treatment from the beginning [6], as the goal is to develop an optimised scalable evidence-based treatment. In addition, internet-delivered therapy has the major advantage that treatment content can be standardised and fixed, and written therapist responses can be closely demarcated, reducing unwanted “drift” from treatment protocols [7]. This helps prevent potential contamination between different treatment components, an essential condition for a factorial design.

Consistent with the MOST framework, we chose the treatment components to ensure that they are conceptually and operationally distinct from each other, so that each can be evaluated independently. This does not mean that we assume that the treatment components do not interact; we will use the factorial approach with effect coding as this allows us to concurrently test both main effects and interactions [14, 17]. The current design assesses the effectiveness of seven treatment components from standard CBT [28] and recent treatment innovations derived from experimental research [29, 30], with each hypothesized to specifically target distinct mechanisms arising from different theoretical models. We will use a fractional factorial design to retain the benefits of a factorial design whilst making the study more logistically manageable and feasible to deliver: the fractional factorial design reduces the total number of conditions from 128 to 32.

IMPROVE-2 builds on a previous feasibility study (IMPROVE-1), which assessed the feasibility of online recruitment of patients with depression, of maintaining treatment integrity and fidelity across randomisation into multiple treatment conditions and of avoiding contamination across treatment conditions, and assessed rates of attrition and retention and treatment adherence (Watkins, Newbold, Cadman, Javaid, Umegaki, Collins, Graham & Mostazir. Implementing multifactorial psychotherapy research in online virtual environments (IMPROVE-1): a feasibility trial of the MOST component selection experiment for internet cognitive-behavioural therapy for depression, In preparation). IMPROVE 1 demonstrated that this approach was feasible, and that it was possible to randomize individuals into multiple treatment conditions (32 in the fractional factorial design) without any contamination and with 100 % fidelity for the interventions received. However, IMPROVE-1 was not powered to test main effects and interactions for each of the seven experimental intervention factors on change in depression symptoms.

### Aims and objectives

The primary aim of this phase III trial is to test the main effects and selected interactions for seven treatment components within internet CBT for depression. The treatment components (in *italics*) and their associated hypothesized mechanisms-of-action are described below.

Within the behavioural model, *activity scheduling* and *absorption training* are hypothesized to increase response-contingent positive reinforcement [31] by respectively increasing frequency of positive reinforcement and direct contact with positive reinforcers. *Functional analysis* is proposed to target habitual avoidance and rumination by identifying antecedent cues, controlling exposure to these cues, and practising alternative responses to them [32].

Within the cognitive model, *thought challenging* is hypothesized to reduce the negative thinking characteristic of depression [28]. *Concreteness training* [30] is hypothesized to specifically reduce the overgeneralization cognitive bias identified as important in depression [33].

Further treatment components are hypothesized to improve emotional regulation. *Relaxation* is proposed to target physiological arousal and tension. *Self-compassion training* is proposed to activate the soothing and safeness emotional system, hypothesized to be downregulated in depression [34–36].

The use of a fractional factorial design will uniquely test hypotheses from different theoretical models concerning the mechanisms-of-action in internet CBT. By comparing the presence versus absence of each component, this factorial design can examine the main effect of each component on the primary outcome (e.g., testing whether thought challenging reduces symptoms of depression) and on secondary outcome measures indexing the specific mechanism theorised to be closely related to each component (e.g., testing whether thought challenging has a main effect on reducing self-reported negative cognition) and, whether this in turn mediates the effect of the treatment component on symptoms. Consistent with the Pareto principle and prior MOST studies [23], we predict that components and interactions will vary in treatment effect size, with many insignificant (i.e., not all components are active in therapeutic benefit of CBT). Watkins and Nolen-Hoeksema (2014) predicted that treatment components that explicitly target depressogenic habits by identifying cueing stimuli and repeatedly training incompatible alternative responses (e.g., functional analysis) will have the largest sustained treatment effect [32].

A further advantage of a factorial design is that it provides a strong test of the relative contribution of specific versus non-specific common treatment factors. An important but as yet unresolved debate concerns the relative contribution of non-specific common factors shared across therapies (e.g., hope, remoralization, therapeutic alliance, treatment credibility) versus specific elements (e.g., reducing negative thinking, increasing behavioural activation). CBT includes generic therapy elements (e.g., providing a rationale, therapist support), and specific elements (e.g., activity scheduling, thought challenging), which are hypothesized to act through different mechanisms. Identifying the relative contribution of specific versus common factors has major theoretical and practical implications for therapy delivery. However, progress at disentangling specific from non-specific treatment effects has been limited because of difficulties in creating psychotherapy placebos (attentional controls) that match the genuine psychotherapy for credibility and structural equivalence [37]. In this factorial design, for any treatment component (e.g. relaxation), the aggregate of the 16 conditions where it is present (i.e., Table 1, conditions 17-32) are equivalent for treatment credibility, structure, delivery, rationale, therapist contact, and therapist allegiance with the aggregate of the 16 conditions where it is absent (i.e., Table 1, conditions 1–16). Hence, the evaluation of the main effect of relaxation involves the comparison of the average effect for the conditions where relaxation is present versus for the conditions where relaxation is absent. This design therefore provides an improved psychotherapy attentional control condition able to disentangle specific from non-specific common treatment factors. A significant main effect for any component would be evidence for a specific treatment effect within CBT for depression beyond non-specific common therapy factors.

**Table 1:** Experimental groups of the fractional factorial design

Condition	Functional Analysis	Concrete training	Compassion	Absorption	Relaxation	Activity Scheduling	Thought Challenging
1	no	no	no	no	no	yes	yes
2	yes	no	no	no	no	no	no
3	no	no	yes	no	no	no	no
4	yes	no	yes	no	no	yes	yes
5	no	no	no	yes	no	yes	no
6	yes	no	no	yes	no	no	yes
7	no	no	yes	yes	no	no	yes
8	yes	no	yes	yes	no	yes	no
9	no	yes	no	no	no	no	no
10	yes	yes	no	no	no	yes	yes
11	no	yes	yes	no	no	yes	yes
12	yes	yes	yes	no	no	no	no
13	no	yes	no	yes	no	no	yes
14	yes	yes	no	yes	no	yes	no
15	no	yes	yes	yes	no	yes	no
16	yes	yes	yes	yes	no	no	yes
17	no	no	no	no	yes	no	yes
18	yes	no	no	no	yes	yes	no
19	no	no	yes	no	yes	yes	no
20	yes	no	yes	no	yes	no	yes
21	no	no	no	yes	yes	no	no
22	yes	no	no	yes	yes	yes	yes
23	no	no	yes	yes	yes	yes	yes
24	yes	no	yes	yes	yes	no	no
25	no	yes	no	no	yes	yes	no
26	yes	yes	no	no	yes	no	yes
27	no	yes	yes	no	yes	no	yes
28	yes	yes	yes	no	yes	yes	no
29	no	yes	no	yes	yes	yes	yes
30	yes	yes	no	yes	yes	no	no
31	no	yes	yes	yes	yes	no	no
32	yes	yes	yes	yes	yes	yes	yes

*Note*: Every factor occurs an equal number of times at high and low levels (i.e. balanced) and all factors are orthogonal to each other. Each effect estimate involves all 32 of the conditions in Table 1, thereby maintaining the power associated with all participants. This Resolution IV design means that all main effects are aliased with 3-way and higher interactions, and all 2-way interactions are aliased with 2-way and higher interactions, on assumption that non-negligible 3-way interactions are unlikely. In contrast, a standard RCT is aliased for all main effects and interactions of treatment components

## Methods

### Study design

A single centre, stratified (moderate depression, operationalized as PHQ-9 score < 20; severe depression, operationalized as PHQ-9 score > =20; source of recruitment: directly from the internet versus within an National Health Service Improving Access to Psychological Therapies (IAPT) treatment service; antidepressant use: none versus at a recommended clinical dose), block randomized, single-blind, fractional factorial trial, based in England will be conducted on the internet, randomized at the level of the patient. It will be a full scale internet-delivered component selection experiment with seven experimental factors evaluated, each at two levels ((presence, coded as +1 versus absence, coded as -1 of component; in other words, effect coded), using a 32-condition balanced fractional factorial design (2_IV_^7-2^)). This design will allow the estimation of all main effects and several pre-specified 2-factor interactions among the seven intervention factors; in statistical terminology, a Resolution IV design because main effects are aliased with 3-way interactions. Table 1 describes the specific combinations of 2-level intervention factors in the experimental design. A full factorial design of seven factors would have required 2^7^ = 128 conditions; the fractional factorial design reduces the total number of conditions by three-quarters.

Within the chosen fractional factorial design, all participants will be randomized to receive at least one component of CBT and in the majority of cases 3 or 4 components of CBT. This design does not have any conditions in which participants receive no treatment components, i.e., there is not a no-treatment, treatment-as-usual, or waiting list control cell, making it suitable for use in a clinical service. Main effects and interactions are estimated based on aggregates across experimental conditions. For each main effect, half of the study population will be randomized to one level of the factor (e.g., conditions 9–16, 25–32 presence of concreteness training) and half will be randomized to the other level of the factor (e.g., conditions 1–8, 17–24 absence of concreteness training); thus, the full sample size can be used to determine each of the main effects (and interactions), making this design efficient for power and sample size.

### Study settings

The study will be conducted over the internet and by telephone with recruitment across all of England. In addition, we will recruit within the IAPT service for the Cornwall Partnership NHS Foundation trust. The intervention will be delivered on the Internet, with the treatment being supported by trained therapists or psychological wellbeing practitioners (PWPs), either based in the University of Exeter or within the IAPT service.

### Participant inclusion criteria

Participants will be adults resident in England, registered with a general practice, who (a) meet criteria for clinical depression, as operationalized on the Patient Health Questionnaire-9 (PHQ-9 ≥ 10) [38], a well-validated self-report measure of depressive symptoms; (b) consent to participate in the study; (c) are aged 18 or over; (d) are a fluent English speaker; (e) have home or work access to the Internet and an e-mail account that is used regularly; (f) are not currently enrolled in another psychological therapy for depression or anxiety. Participants currently receiving antidepressant medication will be eligible, provided the dosage has been stable for at least the previous 4 weeks.

In the IMPROVE 1 feasibility study, approximately 40 % of individuals consented to the internet treatment but then never logged onto the treatment or ceased the intervention before completing the first module. To avoid this loss of power and to optimise the number of treatment components completed, which is central to our primary question of which components are active, we will only randomise eligible participants who complete an initial introductory welcome internet treatment module. This extra step will select for participants who are motivated and engaged with internet CBT and afford participants the opportunity to try out the internet treatment approach and determine if it suits them before committing to the trial.

### Participant exclusion criteria

Participants will not be excluded for any other Axis I and II diagnosis that may be co-morbid with depression e.g., anxiety disorders, eating disorders, personality disorders. Participants with self-reported persistent self-injury, current drug and alcohol problems, mania, paranoia, delusions and hallucinations and active suicidality will be excluded from the trial and signposted to other online (e.g., Samaritans, befrienders) and NHS clinical support services, and directed to their general practitioner.

### Recruitment procedure

There will be two main recruitment pathways. The first pathway will be through direct self-referral by potential participants to the trial, principally via the internet. The recruitment strategy will include websites, a Facebook page, advertisements in social media, posters, flyers, web-links to local and national depression support sites and charities, articles in local newspapers, circulars through large employers, and press releases. Participants who are interested in a trial of free online cognitive behavioural therapy for depression will be directed to an online mood screening website [https://mdcdepressionscreening.ex.ac.uk/ ], which will provide further information on the study and which includes online questionnaires organised in a conditional logical flow to assess key eligibility criteria, such as depression (PHQ-9), suicidal ideation, current treatment, and potential exclusion criteria. Automated feedback will inform participants whether they are eligible for further screening, for example, whether they meet the study criteria for elevated symptoms of depression (PHQ-9 ≥ 10). Individuals presenting with elevated suicidal ideation will be automatically advised to see their GP and provided with contact details for organisations offering support and advice. Eligible participants are invited to leave their contact details (name, telephone number, email address) as consent to be contacted. Those participants who pass the screening phase and indicate an interest in the study will be contacted for a more detailed telephone interview to further explain the study and confirm eligibility. At this stage, the full information sheet and consent form are sent by email to the participant.

The second recruitment pathway will be through the NHS IAPT service for Anxiety and Depression in Cornwall where the internet intervention will be offered as part of the standard treatment protocol for depression. For all referrals to the service, Psychological Wellbeing Practitioners (PWPs) conduct a standardized telephone or face-to-face assessment to confirm that the service is suitable for the patient’s needs, which includes the PHQ-9. At the first available opportunity after screening is complete, including within this first assessment session, patients scoring PHQ-9 > 10 who appear suitable for IMPROVE 2 trial will be asked if they would like internet CBT and to participate in research conducted by the Trust with the University of Exeter. Participants indicating an interest in the trial will have their contact details passed to the research team who will contact them to explain the study and proceed to the baseline interview.

All participants who are eligible and consenting will receive access, free of charge, to the internet CBT platform via the web. Those who are eligible and consent to participate will be sent an URL link to the treatment platform and after logging in set their own security password. Specific intervention components received by participants will vary according to the experimental condition to which they were assigned.

### Baseline and consent procedure

#### Telephone screening and baseline assessment

The baseline screening interview consists of brief screening questions for alcohol and drug use, symptoms of bipolar disorder and psychosis (Psychosis Screening Questionnaire, PSQ [39]), assessment of any relevant past or current treatments, and the Structured Clinical Interview for DSM-IV (SCID-I; [40]) sections on current and past depressive episodes, dysthymia, and generalised anxiety disorder. In addition, questionnaires, delivered either over the telephone or through an online survey or by email or post, will be used to assess levels of worry and rumination [41, 42], and symptom severity of depression (PHQ-9) and generalized anxiety (GAD-7 [43]). Any risk reported during the interview will be assessed using a well-established protocol to ensure appropriate clinical support is obtained. Interviews will be audio-recorded, with the participant’s consent, so that diagnostic status can be independently rated.

#### Consent to trial

**Fig. 1:**
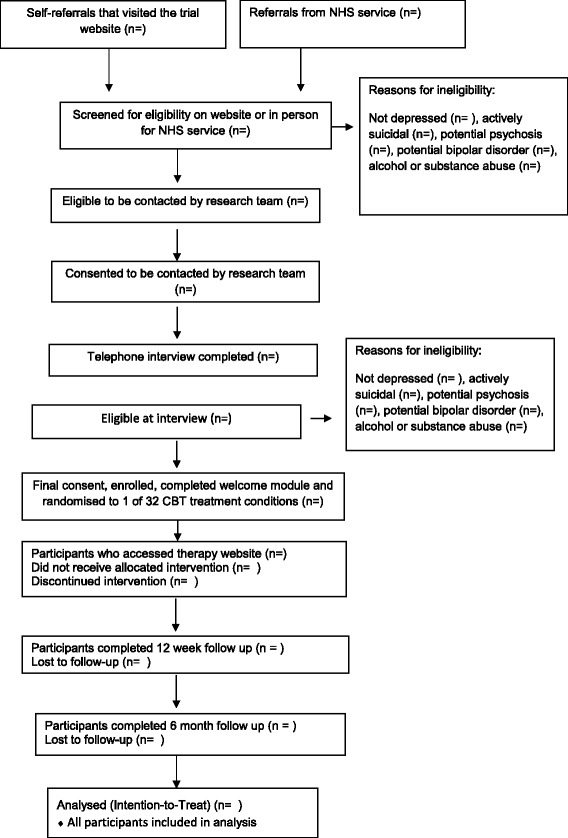
Consort Flow Diagram for IMPROVE-2 study showing recruitment flow

All eligible participants will then be invited to enter the trial and asked to return written consent to participate. Once consent is received and the welcome treatment module is completed, participants will be randomised (see Fig. 1). People who do not fulfil the eligibility criteria will be thanked for their time. Excluded participants are given feedback including advice to see their GP and contact details of organisations for further support, where relevant.

### Randomisation and allocation concealment

Randomisation will be conducted by an independent off-site statistician based within the Peninsula Clinical Trials Unit using computer generated list of quasi-random numbers. Participants will be randomised with equal probability to the 32 conditions. Randomisation will be stratified according to severity of current depression (moderate PHQ-9 ≥ 10 and < 20 vs. severe PHQ-9 ≥ 20), antidepressant use (receiving a BNF recommended therapeutic dose versus not receiving a recommended dose) and by source of referral (NHS referral vs direct internet referral). All outcome assessors and data analysts will be kept blind to treatment allocation. The therapists will only have contact with the participant during the intervention and will record the outcome data and the nature and level of assistance given to each participant during each module. In order to preserve blinding of the study researcher, randomisation will be conducted by a third party not involved in assessing or treating the participants, who will also inform the therapist, who will be responsible for informing participants of their allocation.

### Sample size calculation

We assumed that the smallest Meaningful Clinical Important Difference (MCID) would be a small effect size (Cohen’s d or standardized mean difference = .2) for the main effect of an individual treatment component or interaction between components on pre-to-post change in depression. Any smaller effect would be of little clinical interest or value. In order to detect a MCID of d = 0.20 with 80 % power at α = 0.10 per treatment component/interaction for pre-post change on depression, we require a sample size of N = 632 (NQuery 7.0). Alpha of 0.1 is recommended for component selection experiments to decrease the relative risk of Type II to Type I error when selecting treatment components; i.e., to avoid prematurely ruling out potentially active treatment components [14, 17]. Conservatively estimating 40 % dropout attrition post-treatment, we require N ≈ 1056 for a traditional ANOVA. However, because there are at least five repeated measures on the primary outcome within the treatment, we can use a latent growth curve model, using mean scores as in ANOVA, which typically requires 30-50 % fewer participants for the same power [44]. Conservatively estimating that the use of growth curve modelling effectively reduces our required sample size by 30 % relative to only using first and last time-point as in ANCOVA, we require N ≈ 736.

### Intervention component factors

The content of the program will be based on CBT for depression, programmed by the research team [https://mindresolve.minddistrict.co.uk] hosted on an established and secure internet-treatment platform provided by MindDistrict, a Dutch internet health services company. All participants will complete a welcome module which includes a mood diary and basic psychoeducation about depression prior to randomisation and then if randomised will receive between 1 to 7 treatment components depending on the randomized treatment condition.

Each module takes around an hour to complete in session and 1-to-2 weeks to practise. Each treatment component will include written psycho-education, images, written vignettes, online experiential exercises using audio-recordings with automatic feedback, downloadable audio exercises to practice skills, interactive questionnaires, video vignettes of patients explaining therapy, and response boxes in which patients write about their experiences and plans. The modules each follow the same basic structure: reflection on previous session; introduction of new technique; practical exercises and planning how to practise or implement the technique in daily life. The intervention is accessed through a secure website, with each participant having a password protected account. Participants’ log-ins will be automatically recorded by the programme, allowing for an automated measure of treatment compliance. Reminder emails will be sent to participants after two weeks if they have not completed the module. The participant can work through each module at his own pace but can only move from one module to the next once the therapist has provided feedback.

All components will involve brief prescribed therapist online support to improve retention and adherence [7], in which secure online written feedback is provided at the end of each completed module, with the option for additional secure messaging between therapist and patient. The coach will highlight any positive steps made and encourage participants to sustain these as well as pointing out areas to focus on over the next module. Participants will also be able to send questions to their assigned therapist throughout the programme if they are having difficulty with a specific exercise. Well-established self-report measures of anxious and depression symptoms (PHQ-9, GAD-7) are presented at the end of each component to assess symptom change.

### Functional analysis

Functional analysis (FA) seeks to determine the functions and contexts under which desired and unwanted behaviours do and don’t occur and, thereby, find ways to systematically increase or reduce these behaviours, by exploring their antecedents, consequences, and variability, and then either alter the environment to remove antecedent stimuli that trigger unwanted behaviours and/or practice incompatible and constructive alternative responses to these antecedents. This approach is based on Behavioural Activation (BA) [45, 46] and rumination-focused CBT [29, 47] approaches to depression. Within this module, patients complete questionnaires to identify their warning signs for stress and rumination and to explore the consequences of their behaviour, make plans to alter environmental contingencies that maintain stress, and generate “If Then” implementation intention plans to act differently to those warning signs.

### Relaxation

Within this module, a variant of progressive muscle relaxation and breathing exercises is used to reduce physiological arousal and tension in response to warning signs, based on evidence that this intervention alone reduces depression [30]. The module introduces a rationale for relaxation, provides an online relaxation exercise as a behavioural experiment to test if it reduces tension, and a downloadable relaxation exercise.

### Identifying and challenging negative thoughts

This module follows the standard steps within CBT for depression as outlined in the 1979 Beck manual [28], including psychoeducation about negative automatic thoughts and cognitive distortions, vignettes of identifying and challenging negative thoughts, and written exercises in which patients practise identifying and then challenging negative thoughts using thought records.

### Activity scheduling

This module closely follows the activity scheduling element of CBT for depression [28]. The internet module provides psychoeducation about the negative effects of avoidance, includes questionnaires to help patients identify their own patterns of avoidance, provides guidance on activity scheduling to build up positive activities and reduce avoidance (e.g., breaking plans into smaller steps; specifying when and where to implement activities), and exercises in which participants generate their own activity plans.

### Concreteness training

This module is based on the concreteness training intervention found to reduce symptoms of depression [30] and derived from experimental research indicating the benefits of shifting into a concrete processing style [48, 49]. It provides psycho-education about depression, rumination, and overgeneralization, a behavioural experiment using audio-recorded exercises to compare abstract versus concrete processing styles, and downloadable audio exercises to practise thinking about negative events in a concrete way. In these exercises, patients identify a recent mildly to moderately upsetting difficulty and work through standardized steps: (i) using mental imagery to focus on sensory details during the difficult event, noticing what is specific about the event and the context in which it occurs; (ii) noticing the process and sequence by which the difficult event unfolds (‘How did it happen?’); (iii) focusing on how to move forward by specifying the particular steps and behaviours to do next.

### Absorption training

Absorption training is focused on teaching an individual to mentally engage and become immersed in what she is doing in the present moment to improve her direct connection with experience and enhance contact with positive reinforcers. Within the module, patients complete a behavioural experiment using audio-recorded exercises to compare visualisations of memories of being absorbed versus not being absorbed in a task, learn about flow [50], practice generating a more absorbed mind-set using downloadable audio exercises, and identify absorbing activities.

### Self-compassion training

Recent research has highlighted the potential benefit of increasing self-compassion in treatments for depression [29, 35, 36, 51]. Within this module, patients read psychoeducation about compassion including useful self-statements to encourage and support oneself, complete a behavioural experiment that compares their own self-talk to how they talk to others, try an audio-recorded exercise visualising past experiences of self-compassion to activate this mind-set and test its benefits, which is downloadable for further practice, and identify activities they would do more of and activities they would do less of to be kinder to themselves.

The internet therapy will be provided by clinical psychologists or qualified PWPs given specific training in the internet CBT treatment approach. To ensure treatment integrity and fidelity, there will be (a) 32 distinct programmed packages, one for each treatment condition: each patient can access only the specific package allocated, constraining patient and therapist responses to the relevant treatment protocol and ensuring identical content for all participants in each condition; (b) scripted templates for therapist responses for each module provide the coach with constrained feedback faithful with the treatment model, which they tailor to individual clients’ responses; (c) checklists specifying allowed and prohibited elements for each package; (d) extensive therapist training and weekly supervision to a criterion standard; (e) All responses from both client and therapist are automatically saved by the online platform and a random sample will be checked against the templates for adherence and competence.

### Blinding

This is a single blind study, with the researcher conducting outcome measures blind to allocation. Participants will be asked at the end of the screening interview not to disclose their allocation to the researcher in any of their future correspondence with the researcher and reminded of the importance of this prior to and during each follow-up interview. Due to the intervention, participants and therapists cannot be blinded. Because therapists are not blinded to participant’s treatment, appropriate clinical steps can be taken (e.g., withdrawing treatment, onward referral) as needed in response to clinical presentation.

### Follow-up assessments and outcome measures

Participants will complete up to three online assessments: a baseline assessment and then at 12 weeks and 6 months after randomisation. Symptoms of depression and anxiety will be assessed at each follow-up, and diagnostic interviews conducted at baseline and at 12 weeks. At follow-ups, participants will be asked about any changes in medication or other treatment that they may have received since they started the trial.

The primary outcome is level of symptoms of depression as assessed by change in Patient Health Questionnaire-9 score (PHQ-9) [38] from randomisation baseline to 12 weeks in the Intention-To-Treat population that involved all patients who were randomly assigned. Secondary outcomes include levels of anxiety as assessed by the Generalized Anxiety Disorder-7 (GAD-7) [43], and social, home and work functioning as assessed by the Work and Social Adjustment Scale (WSAS) [52].

In addition, participants will complete a series of self-report questionnaires designed to capture the primary mechanism which each treatment component is hypothesized to most strongly influence, including rumination (5-item Brooding scale) [41], change in habitual coping (adapted Self-Report Habit Index) [53] (SRHI), overgeneralization (adapted Attitudes to Self Scale – Revised) [54], self-compassion [55], negative thinking (Automatic Thoughts Questionnaire) [56], increased behavioural activity and reduced avoidance (Behavioural Activation for Depression Scale Short-form) [57], and absorption and engagement in positive activities, adapted from measures of “flow” [58]. These variables will be tested in mediational analyses.

Each assessment will take place via a telephone interview, with the option of the questionnaire measures being returned by online electronic survey, email or post. To increase participant retention and completion of follow-ups, multiple attempts and multiple means (email, telephone, post) will be used to contact participants. Additionally, to increase motivation to complete the follow-up measures, lottery draws for online shopping vouchers will be held, with each participant receiving one ticket per completed follow-up. Research data will be collected in an anonymised manner by means of the interviews, online questionnaires or postal paper questionnaires. Questionnaires from respondents who prefer a paper version will be entered into the data system by a research assistant. An administrative database is used to ensure timely assessments. The majority of data will be collected electronically and downloaded automatically to reduce errors and missing data. Information in the internet intervention is password protected, on secure encrypted servers using SSL. Each participant included in the trial will be assigned a unique identifier and all data stored without identifying details. Contact details (name, email, telephone number, GP) are stored separately to enable follow-up contacts and management of clinical risk. Data will be held on a secure database on a password-protected computer. Access to data will be restricted to the research team.

### Statistical analysis plan

Data cleaning will follow the protocol set out by Tabachnick and Fidell [59] including range checks for data values. Statistical reporting will follow CONSORT standards [60]. Missing data will be inspected and handled via full information maximum likelihood (FIML) or multiple imputations (MI) as appropriate. Primary analyses will be conducted on the Intention-To-Treat (ITT) sample. A full, detailed analysis plan, including plans for any interim analysis, subgroup analysis, and sensitivity analysis of the primary outcomes, will be prepared and finalised before the analysis.

The primary outcome is change in PHQ-9 score from randomisation baseline to 12 weeks in the intention-to-treat population that involved all patients who were randomly assigned. Secondary outcomes include GAD-7 and process-mechanism measures.

Our analysis will be an Analysis of Covariance (or equivalent regression or growth curve models) for the primary and secondary endpoints. Each main effect will be modelled as a fixed effect, with baseline PHQ-9 as the covariate. Our interest is primarily in tests of ANOVA main effects and interactions. Main effects and interactions are estimated based on aggregates across experimental conditions (see Fig. 1). Models will be fitted using generalized linear mixed models or latent growth curve modelling, equivalent to ANCOVA regression models, adjusted for stratification variables and baseline outcome values.

Under sub-optimal adherence, subsequent analyses will use the Complier Average Causal Effect (CACE) analysis, which assumes that under true randomization, the probability of non-compliance in those randomized to the absence of a treatment component is the same as that observed in those randomized to the presence of a treatment component, and merely offering the treatment component has no bearing on the outcome, i.e. randomization has no direct effect on outcome but rather outcome depends on the nature of compliance [61, 62]. It therefore provides estimates of a treatment effect taking into account adherence and compliance with the treatment, whilst retaining the benefits of randomisation. It is an example of using an Instrumental Variable (IV) where randomisation is the instrument, which is correlated with compliance to the treatment, and directly unrelated to the outcome. Since randomization will be done to multiple treatment components, which will create several latent classes of compliance, we will use Maximum Likelihood (ML) based Simultaneous Equation Model (SEM) to apply such an IV approach.

### Mediation and moderation analysis

Mediational analyses will be used to test the hypotheses that each treatment component primarily works through the hypothesized mechanism-of-action, using the approach outlined by Kraemer et al. [63]. We will use modern causal inference methods including SEM to test for mediation of the treatment components on depression through changes in negative thinking, avoidance, approach behaviours, rumination, compassion, etc. Analyses will adjust for baseline measures of the mediator, outcomes, and putative measured confounders and non-linear tests along with bootstrapped standard error will be carried out to test the mediation effect. In addition, we will investigate potential moderation of the treatment components by site, age, sex, severity of depression, co-morbid illness and antidepressant use.

### Ethics and Governance

Ethical and professional guidelines will be followed at all times, in line with Good Clinical Practice guidelines. Any possible adverse events witnessed by researchers or the therapists will be discussed as soon as possible with the principal investigator. If this is deemed to be an adverse event, an adverse event report will be completed. In the case of serious adverse events, the University of Exeter, as sponsor, will also be notified using the same report. Any suicidal risk reported during assessment interviews or using the internet platform is assessed using a well-established protocol to ensure that appropriate clinical support is provided. Users of the internet treatment are provided with links to online support, access to secure messaging to trial team, and automatic signposting to help and guidance if reporting risk within the intervention. A Trial Steering Committee has been created with experts in depression, internet treatment and clinical trials, and lived experience representatives, to monitor and supervise the progress of the trial, to provide oversight of the trial, including determining if interim analyses are necessary and evaluating such data, to consider any safety issues for the trial and recommend appropriate contingencies, and to ensure that it is being conducted in accordance with the principle of Good Clinical Practice and the relevant regulations. All patients are able to discontinue the internet treatment by their own choice whenever they wish.

## Discussion

The current trial has been designed to provide the first examination of the underlying active treatment components within internet CBT for depression, by using a factorial experimental design. Understanding the active components of therapy will enhance our understanding of therapeutic mechanisms and potentially enable the systematic building of more effective interventions.

The current trial involves the optimisation stage in the MOST process in which a screening experiment is conducted to identify active components within internet CBT for depression. In further research stages, this information could be used to develop an optimised intervention and to then evaluate its efficacy in a comparative randomised controlled trial with existing recommended treatments. If this approach indicates that some but not all components within internet CBT for depression have a significant effect size in reducing depression, it will lead to the building of better therapies that focus on the active ingredients and discard inert or iatrogenic elements. We believe that this innovative approach may provide a useful means to address recent requests for rigorous study designs to determine which elements within psychological interventions are critical [12].

### Trial status

The trial was registered at Current Controlled Trials ISRCTN number: ISRCTN24117387. Recruitment began in July 2015 and was ongoing at time of submission.

## Additional file

Additional file 1: SPIRIT 2013 Checklist: Recommended items to address in a clinical trial protocol and related documents. (DOC 121 kb)

## Abbreviations

CONSORT: Consolidated standards of reporting trials
GAD-7: Generalized anxiety disorder 7-item scale
GP: General practitioner
ITT: Intention-To-Treat
MI: Multiple imputation
MRC: Medical Research Council
PHQ-9: Patient Health Questionnaire 9 item
PSQ: Psychosis Screening Questionnaire
PSWQ: Penn State Worry Questionnaire
RCT: Randomised controlled trial
RRS: Ruminative Response Scale of the Response Styles Questionnaire
SCID-I: Structured Clinical Interview for DSM-IV
TAU: Treatment-as-usual
